# Promoting active travel to school: a systematic review (2010–2016)

**DOI:** 10.1186/s12889-017-4648-2

**Published:** 2017-08-05

**Authors:** Bo Pang, Krzysztof Kubacki, Sharyn Rundle-Thiele

**Affiliations:** Department of Marketing and Social Marketing @ Griffith, Griffith University, 117 Kessels Road, Nathan, QLD 4111 Australia

**Keywords:** Children, Physical activity, Active school travel, Interventions

## Abstract

**Background:**

Interventions aiming to promote active school travel (AST) are being implemented globally to reverse AST decline. This systematic literature provides an update of AST interventions assessing study quality and theory use to examine progress in the field.

**Methods:**

A systematic review was conducted to identify and analyse AST interventions published between 2010 and 2016. Seven databases were searched and exclusion criteria were applied to identify 18 AST interventions. Interventions were assessed using the Active Living by Design (ALBD) Community Action (5P) Model and the Evaluation of Public Health Practice Projects (EPHPP). Methods used to evaluate the effectiveness of each intervention and their outcomes and extent of theory use were examined.

**Results:**

Seven out of 18 studies reported theory use. The analysis of the interventions using the ALBD Community Action Model showed that Preparation and Promotion were used much more frequently than Policy and Physical projects. The methodological quality 14 out of 18 included interventions were assessed as weak according to the EPHPP framework.

**Conclusion:**

Noted improvements were an increase in use of objective measures. Lack of theory, weak methodological design and a lack of reliable and valid measurement were observed. Given that change is evident when theory is used and when policy changes are included extended use of the ALBD model and socio-ecological frameworks are recommended in future.

**Electronic supplementary material:**

The online version of this article (doi:10.1186/s12889-017-4648-2) contains supplementary material, which is available to authorized users.

## Background

Active school travel (AST) remains an important source of physical activity for children [1]. AST has been shown to provide benefits such as reduction in children’s Body Mass Index that long-term leads to a reduction in obesity-related diseases [2], improvement in academic performance at school [3], and as part of a larger picture, reduction in car use benefitting the environment [4]. Compared with other forms of physical activity, AST has the additional advantage of being convenient and free of monetary costs [5]. However, there is evidence that AST has significantly declined over the past 30 years [6, 7]. Studies investigating the reasons behind the decline in AST point towards increasing use of car transportation, change in social norms [8], and parental concerns about safety (e.g. abduction, traffic, crime, and strangers) [9] as key contributors to the decline, amongst other factors.

Behavioural change interventions have attempted to reverse the decline in AST. For example, a systematic review by Chillon and colleagues [10] identified 13 interventions reporting a trivial to strong positive impact on AST behaviour. However, opportunities for improvement in future studies were identified including measurement, methodology and use of theory in intervention design and/or evaluation. A review update to consider progress in the field is timely to extend understanding.

Systematic literature reviews offer two key benefits. Firstly, systematic literature reviews guarantee that a more reliable knowledge base can be developed without biases that can occur in narrative reviews [11, 12]. Secondly, systematic literature reviews can inform policy makers and practitioners by reporting the effectiveness of interventions [12]. Therefore, the purpose of the current study is three-fold. First, we aim to conduct a systematic literature review and analysis of AST interventions published between 2010 and 2016. Second, we compare the results of our review with Chillon et al. [10] to assess whether significant differences in theory use, measurement and design are evident between time periods. Third, we assess the extent of theory use for AST interventions reporting theory.

## Method

### Data source and search strategy

This study followed Chillon et al. [10] search terms and systematic literature review procedures (see procedures outlined in13,14) to identify peer-reviewed journal articles reporting AST interventions published between 2010 and 2016. Seven databases (EBSCO All databases, Emerald, ProQuest All databases, Ovid All databases, ScienceDirect, Taylor & Francis, and Web of Science) were searched using the following terms:

1. active transport* OR active travel*
2. intervention* OR Randomi?ed Controlled Trial OR evaluation OR trial OR campaign* OR program* OR study OR studies
3. child* OR adolescent* OR parent* OR youth OR student* OR pupil*
4. school*

The symbols ‘*’ and ‘?’ are used as wildcards to include possible plurals and American/British spelling versions of the relevant terms respectively. The search terms were determined by multiple experiments using different combinations of terms in database searches to maximize the likelihood of retrieving the most relevant results. The seven databases used in this review were selected as they include marketing- and health-oriented publications and these were consistent with databases reported in previous systematic literature reviews [13, 14]. An additional file summarises the search strategy [see Additional file 1]. The numbers of articles retrieved from each database are shown in Table 1 below:

**Table 1:** Numbers of articles retrieved

Database	Number of articles retrieved
EBSCO All Databases	149
Emerald	6
ProQuest All Databases	287
Ovid All Databases	322
ScienceDirect	2
Taylor & Francis	130
Web of Science	657
Total	1553

### Exclusion criteria

A total of 1553 records were identified in the search. All records were downloaded and imported into EndNote. After removal of all duplicated records (*n* = 696), 857 unique records were then checked against the following exclusion criteria to remove unqualified records:

1. not peer-reviewed journal articles, ensuring that all included sources had been peer-reviewed. Other types of records such as magazines, conference proceedings, newspapers, and dissertations were excluded;
2. not in English;
3. not related to AST;
4. policy related articles;
5. review/conceptual articles;
6. articles containing only formative research;
7. medical trials;
8. articles published before 2010.

After application of the exclusion criteria 27 qualified records remained. In the following stage backward and forward searches were conducted including examination of all reference lists of the 27 articles and searching authors’ names and websites, and intervention names in Google Scholar. A further 13 articles providing additional information about already identified AST interventions and one additional new intervention were identified. The process produced a total of 40 peer-reviewed articles published between 2010 and 2016 reporting a total of 18 AST interventions. PRISMA guidelines [15] were followed to systemically analyse the articles and report our review.

**Fig. 1:**
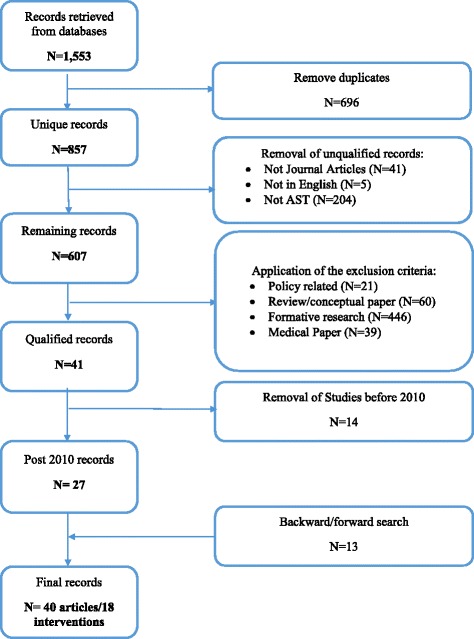
The systematic review process

Figure 1 demonstrates the search process, and a full list of 40 papers for each intervention can be found in the Appendix.

### Data analysis

The following data was extracted and analysed from the papers:

1. Intervention strategy. In line with the method employed in Chillon et al.’s review [10], the Active Living by Design (ALBD) Community Action (5P) Model was adopted to analyse the strategies used in the interventions. This framework consists of five components: 1) Preparation, which includes “developing and maintaining a multidisciplinary community partnership, collecting relevant assessment data to inform program planning, providing relevant training, and pursuing financial and in-kind resources to build capacity” [16] (p. 315); 2) Promotion, which refers to engaging the target audience with dedicated messages and materials; 3) Program, which refers to ongoing organised activities that aim to engage individuals; 4) Policy, which refers to rules or standards that are set to regulate behaviours; and 5) Physical projects, which refer to environmental changes that are made to remove barriers to physical activity. Additionally, reported theory use was extracted and analysed as it has been linked to enhanced intervention outcomes [17], and theory use in AST interventions was previously found to be lacking [10]. The framework of assessing theory utilization was used in previous systematic reviews [18, 19]. The framework consists of four levels, namely 1) Informed by theory, which means theory was identified but no or limited application of theoretical framework was used; 2) Applied theory, which means several components and measures were applied in the study; 3) Testing theory, which means more than half the theoretical constructs were explicitly measured and tested, or there exists theory comparison; 4) Building theory, which means revising or creating theory by measuring, testing, and analysing constructs.
2. Intervention design and delivery. The Evaluation of Public Health Practice Projects (EPHPP) [20] was adopted to assess the quality of the interventions and ensure consistency in reporting with earlier research [10]. EPHPP was developed to provide research evidence to support systematic intervention reviews by outlining step-by-step guidelines [21]. EPHPP has been used in a wide range of content areas, such as chronic disease prevention [22], family health [23], and substance abuse prevention [24]. EPHPP assesses six aspects of interventions: selection bias, study design, confounders, blinding, withdrawals and drop-outs, all of which is synthesised to calculate a global study rating. In EPHPP, each of the aspects are rated on a three-point scale, and the final global rating is based on the rating of the six aspects and identified as strong, moderate, or weak, based on the EPHPP guidelines [20].
3. Evaluation methods and outcomes. In contrast to the methods reported in Chillon et al. [10], who calculated the effect size for each intervention using Cohen’s d, in this study we only extracted the information about the methods used to evaluate the effectiveness of each intervention and their outcomes as reported in the papers. Although Cohen’s d could be an indicator of the effect size of the interventions, the identified heterogeneity of outcome measures among interventions makes the effect sizes incomparable with each other.

All data were extracted from the articles by two independent researchers and the final data were compared and verified to ensure accuracy. Discrepancies were minor and were resolved by discussion with a third researcher. In order to compare our results with Chillon et al.’s [10], we adopted the Fisher’s exact test to calculate the *p* value. Fisher’s exact test has been widely used to compare differences among small samples [13], and can offer more accurate results than the conventional Chi-Square method [25].

## Results

### Intervention overview

Forty articles were identified in this review, covering 18 AST interventions. Full intervention details can be found in Table 2.

**Table 2:** AST intervention summary

No.	Intervention	Location	Intervention Year	Intervention Length	Sample Size	Study Aim
1	Beat the Street [42]	UK/Canada	Not Given	4 weeks	3817	AST*
2	DOiT [32]	Netherlands	2003-2004	20 months	1108	HE**, PA***
3	Drop-Off [33]	Belgium	2013	4 weeks	58	AST
4	Health In Adolescents [34]	Norway	2007-2009	20 months	3857	HE, PA
5	Healthy Homework [39]	New Zealand	2009	6 weeks	97	PA, HE
6	It’s Your Move! [38]	Australia	2006-2008	3 years	3040	HE, PA
7	Nevada Moves Day [26]	US	2012	2 months	1336	AST
8	Ride2School [7]	Australia	2006-2010	2 years	13 schools	AST
9	Safe Routes to School - Eugene [27]	US	2007-2011	5 years	16,500	AST
10	Safe Routes to School – Hawaii [28]	US	Not Given	6 months	13 schools	AST
11	Safe Routes to School - Texas [29]	US	2009-2012	4 years	78 schools	AST
12	School Travel Planning - Canada [41]	Canada	2007-2011	3 years	5423	AST
13	School Travel Plans - New Zealand [40]	New Zealand	2004-2008	4 years	57,096	AST
14	Stockholm County Implementation [35]	Sweden	2009-2011	2 years	831	HE, PA
15	Traveling Green [37]	UK	2008 - 2009	18 months	166	AST
16	Trygog Sikker Skolecykling [36]	Denmark	2010-2011	1 year	2401	AST
17	Walking School Bus - Houston [30]	US	2009	5 weeks	149	AST
18	Walking School Bus - Missouri [31]	US	2007	2 months	400	AST

*AST: Active school travel

**HE: Healthy Eating

***PA: Physical Activity

All interventions were conducted in developed countries – including the United States (*N* = 6) [26–31], Europe (*N* = 6) (1 in Netherlands [32], 1 in Belgium [33], 1 in Norway [34], 1 in Sweden [35], 1 in Denmark [36], and 1 in the UK [37]), Australia (*N* = 2) [7, 38], New Zealand (*N* = 2) [39, 40], Canada (*N* = 1) [41], and both the UK and Canada (*N* = 1) [42]. All of the interventions targeted children. The aims of each intervention varied: 13 interventions aimed to promote AST only [7, 26–31, 33, 36, 37, 40–42], and five interventions had multiple aims [32, 34, 35, 38, 39], including promoting healthy eating and physical activity, in which AST only served as one of the physical activity aims. Only three out of 18 interventions conducted pilot tests [31, 37, 41]. The intervention lengths varied from 4 weeks to 5 years, and the sample sizes varied from 58 to 57,096.

### Intervention strategy and theory use

Chillon et al. [10] noted “the studies generally failed to describe their theoretical frameworks” (p. 8), however they did not report whether each of the studies reported or adopted theories. In our review, seven out of 18 interventions reported using theory. The most commonly used theory was Social Cognitive Theory reported (*n* = 5) [29, 30, 34, 39, 42], followed by the Social Ecological Framework reported (*n* = 2) [34, 35] and the Theory of Planned Behaviour (*n* = 2) [37, 39]. Two interventions [34, 39] reported using more than one theory. In terms of theory utilization level, there are two studies [29, 39] informed by theory and two studies [35, 42] which applied theory. For example, in the “Beat the Street” [42] intervention, the researchers introduced competition to win points if children walk to school, underpinning social cognitive theory and learning theory. Three studies [30, 34, 37] tested theory. For example, in the “Traveling Green” [37] intervention, the factors of the Theory of Planned Behaviour were measured and tested to explain active commuting. None of the studies built theory.

All interventions were analysed using the ALBD Community Action Model (see Table 3). Three interventions included all five strategies from the Community Action Model [36, 40, 41]. Five interventions included four strategies, three of which did not implement policy [7, 27, 29], and two did not implement physical projects [35, 38]. Fisher’s exact tests were used to compare the strategies used in the Chillon et al. review [10] and our review and none of the 5Ps were significantly different.

**Table 3:** Intervention Stage

Intervention	Intervention Strategy						Behavioural Change Theory used	Theory utilization level
	Preparation	Promotion	Policy	Physical Projects	Program	No. of Ps		
School Travel Planning - Canada [41]	✓	✓	✓	✓	✓	5	Not Given	n/a
School Travel Plans - New Zealand [40]	✓	✓	✓	✓	✓	5	Not Given	n/a
Trygog Sikker Skolecykling [36]	✓	✓	✓	✓	✓	5	Not Given	n/a
Ride2School [7]	✓	✓	x	✓	✓	4	Not Given	n/a
Safe Routes to School – Texas [29]	✓	✓	x	✓	✓	4	Social ecological model, and social cognitive theory	Reported theory
Safe Routes to School - Eugene [27]	✓	✓	x	✓	✓	4	Not Given	n/a
It’s Your Move! [38]	✓	✓	✓	x	✓	4	Community capacity framework	
Stockholm County Implementation [35]	✓	✓	✓	x	✓	4	Social-ecological model	Applied theory
Beat the Street [42]	✓	✓	x	x	✓	3	Learning theory and social cognitive theory	Applied theory
DOiT [32]	✓	✓	x	x	✓	3	Not Given	n/a
Drop-Off [33]	✓	x	✓	x	✓	3	Not Given	n/a
Health In Adolescents [34]	✓	✓	x	x	✓	3	Social ecological framework, social cognitive theory	Tested theory
Safe Routes to School - Hawaii [28]	✓	✓	x	x	✓	3	Not Given	n/a
Walking School Bus - Houston [30]	✓	✓	x	x	✓	3	Social cognitive theory	Tested theory
Healthy Homework [39]	x	✓	x	x	✓	2	Theory of reasoned action; Theory of planned behaviours; social cognitive theory	Reported theory
Nevada Moves Day [26]	x	✓	x	x	✓	2	Not Given	n/a
Traveling Green [37]	x	✓	x	x	✓	2	Theory of planned behaviours	Tested theory
Walking School Bus - Missouri [31]	✓	x	x	x	✓	2	Not Given	n/a
Use of ALBD in Chillon et al. (2011)	92%	85%	23%	38%	92%			
Use of ALBD in current review	83%	89%	33%	33%	100%			

### Quality assessment

The quality assessment of identified interventions was next conducted using the EPHPP tool^1^ (see Table 4). Two researchers independently assessed all relevant articles and only minor discrepancies were identified and later resolved by discussion with the third researcher. Fourteen studies were assessed as weak in the global rating. None were assessed as strong. Comparing with the results reported in Chillon et al. [10], in which all 14 included studies were assessed as weak, a minor improvement was observed with four studies in the current review evaluated as moderate [29, 31, 38, 39].

**Table 4:** Quality assessment of included interventions

Intervention	A. Selection Bias (Q1)	A. Selection Bias (Q2)	A SCORE	B. Study Design	B SCORE	C. Confounders (Q1)	C. Confounders (Q2)	C SCORE	D. Blinding (Q1)	D. Blinding (Q2)	D SCORE	E. Data Collection Methods (Q1)	E. Data Collection Methods (Q2)	E SCORE	F. Withdrawals and Drop-outs (Q1)	F. Withdrawals and Drop-outs (Q1)	F SCORE	Global Rating
Healthy Homework [39]	4	3	*	3	**	2	n/a	***	3	3	**	1	3	**	2	5	**	**
It’s Your Move! [38]	4	5	*	3	**	2	n/a	***	3	1	**	1	3	**	1	2	**	**
Safe Routes to School – Texas [29]	3	5	*	3	**	2	n/a	***	3	1	**	1	1	***	2	1	***	**
Walking School Bus - Missouri [31]	3	3	*	3	**	2	n/a	***	3	3	**	1	1	***	4	5	**	**
Beat the Street [42]	3	1	*	5	**	3	4	*	1	1	*	2	3	*	1	3	*	*
DOiT [32]	4	1	*	1	***	1	3	*	1	3	**	1	1	***	1	1	***	*
Drop-Off [33]	3	3	*	6	**	3	4	*	1	1	*	1	3	**	2	1	***	*
Health In Adolescents [34]	3	3	*	1	***	2	n/a	***	3	3	**	1	1	***	2	3	*	*
Nevada Moves Day [26]	3	1	*	3	**	2	n/a	***	3	3	**	3	3	*	4	4	*	*
Ride2School [7]	3	5	*	5	**	3	4	*	1	1	*	3	3	*	2	1	***	*
Safe Routes to School - Eugene [27]	3	4	*	7	*	3	n/a	*	3	3	**	1	3	**	4	4	*	*
Safe Routes to School - Hawaii [28]	3	5	*	3	**	1	4	*	3	3	**	1	1	***	4	5	**	*
School Travel Planning - Canada [41]	3	4	*	5	**	3	4	*	3	3	**	1	1	***	1	3	*	*
School Travel Plans - New Zealand [40]	4	3	*	7	*	3	4	*	3	3	**	1	1	***	3	4	*	*
Stockholm County Implementation [35]	3	3	*	3	**	1	3	*	1	1	*	1	1	***	2	1	***	*
Traveling Green [37]	3	5	*	3	**	3	4	*	3	3	**	1	1	***	3	4	*	*
Trygog Sikker Skolecykling [36]	4	5	*	3	**	1	3	*	3	1	**	3	3	*	2	1	***	*
Walking School Bus - Houston [30]	3	5	*	1	*	1	3	*	3	3	**	1	1	***	2	1	***	*

* = weak; ** = moderate; *** = strong

None of the studies reported representative sampling methods, which resulted in weak scores in category A - selection bias. In terms of study design, three studies reported using randomised control trial design [30, 32, 34] and were therefore assessed as strong; 13 studies were assessed as moderate, with nine cohort analytic (two groups pre + post) [26, 28, 29, 31, 35–39], three cohort (one group pre + post) [7, 41, 42], and one interrupted time series design [33]. It is noteworthy that study [33] self-identified as quasi-experiment with pre- and post-tests, measuring effects during the intervention, therefore the study was classified as interrupted time series. Two studies were rated as weak, including one longitudinal study [40]. Although one study [27] self-identified as quasi-experimental design, no evaluation was reported and therefore the study was assessed as weak. In terms of confounders, six studies reported confounders and no major differences were found between groups, which resulted in a strong rating. The rest of the studies did not report accounting for confounders or had only one group in the design and were therefore assessed as weak.

None of the studies reported to be double blinded. Four studies were assessed as weak as neither assessors nor participants were blinded [7, 33, 35, 42]. The rest of the studies were rated as moderate with either one-directional blinding or no relevant information reported. In terms of data collection methods, ten studies provided evidence of both reliability and validity and thus were assessed as strong [28–32, 34, 35, 37, 40, 41]. Four were assessed as moderate with either reliability or validity being reported [27, 33, 38, 39], and five were assessed as weak as they did not report reliability or validity [7, 26, 36, 42]. Regarding the drop-out rate, seven studies were assessed as strong with more than 80% of participants completing the studies [7, 29, 30, 32, 33, 35, 36]. The remaining studies were assessed as moderate (*n* = 4) [28, 31, 38, 39] or weak (*n* = 7) [26, 27, 34, 37, 40–42] due to either low completion rates or not providing enough withdrawal information.

Fisher’s exact tests were used to compare the EPHPP components between the Chillon et al. [10] review and our review.

Table 5 shows that apart from Data Collection Methods (*p* = 0.011) none of the EPHPP components, including the global rating were significantly different.

**Table 5:** Fisher’s exact p in EPHPP items

EPHPP Items	Ratings	Chillon et al. (2011) (*n* = 13)	Current study (*n* = 18)	*P* value
Selection Bias	Weak	85%	100%	0.400
	Moderate	15%	0%	
	Strong	0%	0%	
Study Design	Weak	23%	17%	0.583
	Moderate	54%	72%	
	Strong	23%	11%	
Confounders	Weak	77%	67%	0.076
	Moderate	15%	0%	
	Strong	8%	33%	
Blinding	Weak	31%	22%	0.689
	Moderate	69%	78%	
	Strong	0%	0%	
Data Collection Methods*	Weak	77%	22%	0.011
	Moderate	8%	22%	
	Strong	15%	56%	
Withdrawals and Drop-outs	Weak	69%	39%	0.289
	Moderate	8%	22%	
	Strong	23%	39%	
Global Rating	Weak	100%	78%	0.120
	Moderate	0%	22%	
	Strong	0%	0%	

*p < 0.05

### Post-intervention evaluation and outcomes

A wide range of evaluation methods were reported in the 18 interventions identified in this review. They can be categorised into two main groups: self-reported and objective behavioural measures. Self-reported measures were identified in 14 interventions, and the most common methods included surveys (*n* = 12) [7, 27–30, 33, 36–38, 40–42], interviews (*n* = 2) [35, 38], and diaries (*n* = 1) [39]. Objective behavioural measures were identified in 12 interventions, and the most common methods included accelerometers, pedometer, and geographic information system (GIS) equipment (*n* = 5) [27, 31, 34, 37, 39], BMI monitoring (*n* = 5) [30, 32, 34, 36, 38], and observations (*n* = 3) [26, 28, 30]. Eight studies combined self-reported and objective behavioural measures such as surveys and observations to triangulate and verify intervention effectiveness [27, 28, 30, 36–39, 42]. A comparison with Chillon et al. [10] indicates an increase in more objective assessment measures in recent years: in Chillon et al. [10], all studies used self-reported measurements and only three studies triangulated data including the addition of objective measurements.

All reported evaluation outcomes are summarised in Table 6. Among 18 interventions, six interventions reported some positive effects on AST [26, 27, 29, 38, 40, 42], two mixed effects on AST [7, 41], and five reported no effect [32, 35–37, 39]. Five interventions did not measure AST behaviour [28, 30, 31, 33, 34] reporting other aims. Positive attitude change was reported in four interventions [30, 33, 34, 41]; positive change in BMI was reported in two [32, 38], positive policy change in two [33, 35], knowledge and long-term infrastructure improvement were each reported in three interventions [7, 29, 41], and finally positive healthy eating and general physical activity changes were reported in one intervention each [30, 32].

**Table 6:** Post-intervention evaluation

No.	Intervention	Intervention Evaluation Method		Outcomes	AST Outcomes
		Self-reporting	Non-self-reporting		
1	Beat the Street [42]	Survey, diary*, and focus group	Swipe card	AST(+);	59% of the participants walked more.
2	DOiT [32]	Survey*	BMI	AST(#); HE(+); BMI(+)	No significant intervention effect on active commuting to school was found.
3	Drop-Off [33]	Survey*	n/a	Attitude(+); PA (+/#); Policy (+)	n/a
4	Health In Adolescents [34]	n/a	Accelerometers* and BMI	Attitude(+/#); PA(+)	n/a
5	Healthy Homework [39]	Diary	Pedometers*	AST(#); PA(+)	No significance intervention effect was observed
6	It’s Your Move! [38]	Survey* and interview	BMI	AST(+); BMI(+)	Walking/Cycling to school increased from 61.1 to 64.6 (*p* = 0.01)
7	Nevada Moves Day [26]	n/a	Observation*	AST(+)	AST increased from pre-intervention to National Moving Day at the intervention school.
8	Ride2School [7]	Survey*	n/a	AST(+/−); Infrastructure(+)	AST increased from 47.9% to 49.6% (*p* = 0.125) (Parent report) AST decreased from 51.1% to 48.7% (*p* = 0.029) (Students report)
9	Safe Routes to School - Eugene [27]	Survey*	GIS*	AST(+)	20% increase in walking and a non-significant increase in biking.
10	Safe Routes to School - Hawaii [28]	Survey*	Observation	Not given	n/a
11	Safe Routes to School – Texas [29]	Survey*	n/a	AST(+); Knowledge(+)	Intervention schools have higher active commuting rates both in the morning and in the afternoon than control schools.
12	School Travel Planning - Canada [41]	Surveys*	n/a	AST(#/+); Attitude(+); Infrastructure(+)	No change in AST at a national level. There is a slight increase of AST in 21 schools in the morning.
13	School Travel Plans - New Zealand [40]	Survey*	n/a	AST(+)	From 2004 to 2008, there were significant increases in active transport especially in the second and third year of implementation.
14	Stockholm County Implementation [35]	Interview*	n/a	AST(#); Policy(+)	No intervention effect was found on AST.
15	Traveling Green [37]	Survey*	Accelerometer* and pedometer*	AST(#)	There was no effect found on AST.
16	Trygog Sikker Skolecykling [36]	Survey	BMI	AST(#); BMI(#); PA (#); Traffic injuries (#)	There was no effect found on school cycling trips.
17	Walking School Bus - Houston [30]	Survey*	Observation* and BMI	PA(+); Attitude(+)	n/a
18	Walking School Bus - Missouri [31]	n/a	Accelerometer*	PA(#); Participation(#)	n/a

+ = positive effect; − = negative effect; # = no effect; * = AST evaluation method

## Discussion

The purpose of this review was three-fold. First, we aimed to provide a contemporary review of AST interventions (2010-2016). Second, we aimed to compare the results of our review with Chillon et al. [10] to track progress in the field. Our review indicated that several issues identified by Chillon et al. [10] continue today and that theory use is limited in AST interventions. Third, we assessed theory utilization in AST interventions. We will focus our discussion on three key aspects, namely theoretical, methodological, and empirical.

### Theoretical aspects

Previous research indicated that theory use in intervention design was associated with enhanced intervention outcomes [17], yet the extent of theory utilization had not been examined previously. In our review seven out of 18 studies reported theory use. Detailed examination identified that two were informed by theory, two applied theories, and three tested theories with examples highlighted in the results section. At the optimal level, theory should provide guidance on the constructs that become the strategic focus of a campaign. Moreover, the theory framework should be used to evaluate the intervention pre and post permitting comparisons of key theoretical constructs focussed upon to be made [43]. The importance of theory adoption and implementation in intervention design is advocated by many researchers [44–46]. Consistent with previous studies our results show that theory testing and building remains limited in AST. For example, Painter et al. [47], identified that 69.1% of health behaviour research used theory to inform a study, in 17.9% theories were applied, in 3.6% theories were tested, and only 9.4% of studies involved building/creating theory.

We propose three recommendations for future AST intervention implementation and reporting. Firstly, future studies should use theory to inform intervention development, execution and evaluation, and detail theory use to facilitate its full comparative assessment across multiple interventions. For example, Schuster et al. [48] used the Theory of Planned Behaviour to gain insights to inform an AST intervention. Results of the study indicated that four variables were found to be highly important in distinguishing carers segments, namely distance to school, current walk to/from school behaviour, subjective norms and intentions to increase their child’s walk to school behaviour. Given that theory can increase the effectiveness of interventions [19, 47] extended application of theory in AST interventions is recommended. Research studies have been undertaken to systematically implement, assess, and report theory utilization in health promotion interventions, such as the UK MRC guidelines [49, 50] and the four-step Theoretical Domains Framework [43], and these are recommended to guide future AST intervention design.

Secondly, the theories used in the interventions identified in our review, such as social ecological theory, social cognitive theory and the Theory of Planned Behaviour, have been considered traditionally as behaviour explanation theories [51]. However, as the ultimate purpose of AST interventions is to change behaviours, predictive theories and model testing should be deployed in future to develop theories focussed on behavioural change. Among all three studies in our review that tested theory [30, 34, 37], theoretical examination depended on cross-sectional regressions limiting understanding to explanation rather than causal understanding. AST interventions should embed predictive theory testing involving longitudinal design across multiple time points to simultaneously explore potential behavioural change determinants. This also requires researchers to focus on utilising more causal/predictive methods rather than variance-based explanation methods in future study design, which is consistent with calls to advance theory to examine behaviour change [52].

Social ecological theory, social cognitive theory and the Theory of Planned Behaviour were most frequently reported and this provides a rich avenue for future research. Lu et al. [53] notes that social ecological theory lacks sufficient specificity suggesting additional testing is needed [53] to establish reliable and valid measures. Individual focussed theories such as Theory of Planned Behaviour and social cognitive theory are limited overlooking structural factors (e.g. policy) which limits understanding of how behavioural change can be facilitated [54]. Therefore, we recommended that theories that were specifically developed in the AST context, such as the McMillan model [55] and the Ecological and Cognitive Active Commuting (ECAC) model [56], should be empirically explored in future AST interventions. For example, the ECAC model specifies three levels of determinants, namely policy, neighbourhood, and individual; that are correlated with AST, covering environmental, social, and psychological aspects providing a wider system view. The McMillan model has been shown to be effective among the general population [57] and young adolescents [58], whereas the ECAC model needs to be empirically tested.

### Methodological aspects

The EPHPP framework was used in this review to assess methodological quality. Fourteen out of 18 studies were assessed as weak. Notably, selection biases, lack of double blinding, and not controlling for confounders were key issues identified in both the current and earlier review [10]. While selection biases arise from practical considerations [28, 41] such as recruiting schools to participate in AST, making it difficult for researchers to control in all circumstances, the current study points to the need for large scale funding permitting optimal study design to be achieved. Issues such as controlling for confounders, on the other hand, can be implemented in most AST interventions. Use of statistical methods, such as case-matching sampling [59], MANCOVA [60], and multi-level modelling [61], are recommended for future AST interventions.

Due to the complexity and diversity of intervention aims, evaluation methods and outcome reporting, we were unable to make direct comparisons of effectiveness between reviews. Standardised outcome measures would permit comparisons and meta-analysis to deliver more detailed understanding in the future. In addition, we recommend that objective measurement methods should be carried out in future intervention design – especially given declining monetary cost of equipment (e.g. smart phones and wearable technology that can automatically capture data) [62–64]. Governments or other funding bodies need to call for more rigour in methodological design and measurement in future.

### Empirical aspects

In line with the results reported in Chillon et al. [10], the current review confirmed the heterogeneity of included studies in terms of their length, sample size, and objectives (see Table 2 for details). However, analysis of the interventions indicated that significant room for improvement remains in terms of broader application of intervention activities. The analysis of the interventions using the ALBD Community Action Model showed that Preparation and Promotion were used much more frequently than Policy and Physical projects. Policy implementation and infrastructure improvements remained limited despite documented positive effects [27, 29] indicating policy use may be a necessary condition for effectiveness [53, 65, 66]. Consistent with the theoretical utilization in AST, Physical programs seem to be effective in promoting AST (see for examples [29, 31]). Our findings are consistent with Lu et al. [53]. In their systematic review, Lu et al. [53] found that social ecological theory is widely adopted and can explain factors preventing children’s walking to school. We recommend that intervention designers should incorporate more school and local policies and infrastructural improvement such as crime prevention and traffic control in order to reduce the perceived risk of AST among parents, observed in many previous reviews [67, 68]. Moreover, habit was not identified as a behavioural determinant in any of included studies although transport habit is an important factor in AST [69]. Future intervention designs should consider facilitating long-term support to convert occasional AST behaviour to a habitual behaviour.

Many of the studies embedded compulsory educational workshops and informational sessions into curriculum (e.g., [32, 39, 40]). Evidence shows that curricular-based interventions results in low attendance and are less effective, which may explain drop-out rates observed for studies employing educational workshops and informational sessions [70]. Therefore, we recommend that instead of educating schools, parents, and children using traditional curricular-based strategies, approaches with more audience engagement be adopted. For example, gamification has been drawing increased attention from intervention designers in recent years, and programs such as GOKA [71] and ONESELF [72] have been shown to achieve substantial audience engagement while delivering outcome effects. Future research should test gamification within AST interventions to extend understanding.

### Limitations

This review has several important limitations, many of which represent opportunities for future research. The search parameters used in the current review limit the studies identified. For example, grey literature and studies not in the English literature were not included in this review. Further, all of the 18 interventions were carried out in developed countries, yet physical inactivity among children is a significant challenge in many developing countries [73] suggesting there is an opportunity to extend AST intervention testing geographically.

A range of outcome measures and methods (self-report and non-self-report) were used to assess AST interventions including attitudes, policy, physical activity, active school travel, BMI, knowledge and infrastructure. The diversity of outcome measures prevents meta-analysis from being undertaken. Different evaluation methods limit potential comparisons between interventions. Further, given physical activity self-report data varies when compared to objective measures (non-self-report) despite high correlations with objective forms such as pedometers and diaries [74], biases must be acknowledged [75]. Moving forward, a unified and consistent approach in reporting AST intervention outcomes is needed to enable meta-analysis to be undertaken in future. Standardisation of outcome reporting would permit effect sizes to be calculated enabling comparison between interventions. Future research is recommended to determine whether there is a relationship between EPHPP quality levels and effect size – an understanding that would inform AST practice.

Meanwhile, the analysis presented in this paper is also limited to the information reported in sources identified in the search process. Employment of a standard reporting framework for AST intervention reporting warrants future research attention ensuring that quality assessments take practicalities into account. For example, full blinding procedures such as those advocated in EPHPP may not be feasible in local government and State funded interventions thereby making this assessment component redundant. Such endeavours may assist to standardise reporting and in turn enhance quality assessment exercises informing future intervention development.

## Conclusion

This review has provided a detailed analysis of AST interventions published in peer-reviewed journals between 2010 and 2016. Following systematic literature review procedures’ 18 AST interventions were identified and subsequently analysed. The main findings of our study are:

1. Theory utilization in AST interventions published between 2010 and 2016 is limited. Where theory is used, interventions informed by theory and interventions that apply theory are much more common than theory testing and building.
2. Considering the ALBD Community Action Model, Preparation and Promotion were reportedly used much more frequently than Policy and Physical projects. Given that change is evident where policy changes are made extended use of the ALBD model is recommended (Preparation, Promotion, Program, Policy and Physical projects).
3. Using the EPHPP framework, 14 out of 18 interventions were weak, largely due to selection biases, lack of double blinding, and not controlling for confounders.
4. Finally, an increase in more objective assessment measures in AST interventions published between 2011 and 2016 was observed, in comparison to the rates reported in Chillon et al. [10].

Issues such as weak methodological design and lack of reliable and valid measurements continue to persist in reported AST interventions, all of which indicate opportunities for further improvements in terms of intervention effectiveness and evaluation.

## Appendix

**Table 7:** List of included papers

No.	Intervention	References
1	DOiT (van Nassau et al., 2013) [32]	**van Nassau F,** Singh AS, van Mechelen W, Paulussen TG, Brug J, Chinapaw MJ: Exploring facilitating factors and barriers to the nationwide dissemination of a Dutch school-based obesity prevention program “DOiT”: a study protocol. BMC Public Health 2013, 13(1):1.
		van Nassau F, Singh AS, van Mechelen W, Brug J, Paw CA, Mai J: In Preparation of the Nationwide Dissemination of the School-Based Obesity Prevention Program DOiT: Stepwise Development Applying the Intervention Mapping Protocol. Journal of School Health 2014, 84(8):481-492.
		Janssen EH, Singh AS, van Nassau F, Brug J, van Mechelen W, Chinapaw MJ: Test–retest reliability and construct validity of the DOiT (Dutch Obesity Intervention in Teenagers) questionnaire: measuring energy balance- related behaviours in Dutch adolescents. Public Health Nutrition 2014, 17(02):277-286.
		Singh AS, Paw MJCA, Brug J, van Mechelen W: Dutch obesity intervention in teenagers: effectiveness of a school-based program on body composition and behavior. Archives of Pediatrics & Adolescent Medicine 2009, 163(4):309-317.
		Singh AS, Paw MJCA, Kremers SP, Visscher TL, Brug J, van Mechelen W: Design of the Dutch Obesity Intervention in Teenagers (NRG-DOiT): systematic development, implementation and evaluation of a school-based intervention aimed at the prevention of excessive weight gain in adolescents. BMC Public Health 2006, 6(1):1.
2	Drop-Off (Vanwolleghem et al., 2014) [33]	**Vanwolleghem G**, D’Haese S, Van Dyck D, De Bourdeaudhuij I, Cardon G: Feasibility and effectiveness of drop-off spots to promote walking to school. International Journal of Behavioral Nutrition and Physical Activity 2014, 11(1):1.
3	Health In Adolescents (Lien et al., 2010) [34]	**Lien N,** Bjelland M, Bergh IH, Grydeland M, Anderssen SA, Ommundsen Y, Andersen LF, Henriksen HB, Randby J, Klepp K-I: Design of a 20-month comprehensive, multicomponent school-based randomised trial to promote healthy weight development among 11-13 year olds: The HEalth In Adolescents study. Scandinavian Journal of Public Health 2010, 38(5 suppl):38-51.
		Bergh IH, van Stralen MM, Bjelland M, Grydeland M, Lien N, Klepp K-I, Anderssen SA, Ommundsen Y: Post-intervention effects on screen behaviours and mediating effect of parental regulation: the HEalth In Adolescents study–a multi-component school-based randomized controlled trial. BMC Public Health 2014, 14(1):1.
		Grydeland M, Bjelland M, Anderssen SA, Klepp K-I, Bergh IH, Andersen LF, Ommundsen Y, Lien N: Effects of a 20- month cluster randomised controlled school-based intervention trial on BMI of school-aged boys and girls: the HEIA study. British Journal of Sports Medicine 2013:bjsports-2013-092284.
		Grydeland M, Bergh IH, Bjelland M, Lien N, Andersen LF, Ommundsen Y, Klepp K-I, Anderssen SA: Intervention effects on physical activity: the HEIA study-a cluster randomized controlled trial. International Journal of Behavioral Nutrition and Physical Activity 2013, 10(1):1.
		Bergh IH, Bjelland M, Grydeland M, Lien N, Andersen LF, Klepp K-I, Anderssen SA, Ommundsen Y: Mid-way and post-intervention effects on potential determinants of physical activity and sedentary behavior, results of the HEIA study-a multi-component school-based randomized trial. International Journal of Behavioral Nutrition and Physical Activity 2012, 9(1):1.
4	Healthy Homework (Duncan et al., 2011) [39]	**Duncan S,** McPhee JC, Schluter PJ, Zinn C, Smith R, Schofield G: Efficacy of a compulsory homework programme for increasing physical activity and healthy eating in children: the healthy homework pilot study. International Journal of Behavioral Nutrition and Physical Activity 2011, 8(1):1.
5	It’s Your Move! (Mathew et al., 2010) [38]	**Mathews LB,** Moodie MM, Simmons AM, Swinburn BA: The process evaluation of It’s Your Move!, an Australian adolescent community-based obesity prevention project. BMC Public Health 2010, 10(1):1. Millar L, Robertson N, Allender S, Nichols M, Bennett C, Swinburn B: Increasing community capacity and decreasing prevalence of overweight and obesity in a community based intervention among Australian adolescents. Preventive Medicine 2013, 56(6):379-384. Millar L, Kremer P, de Silva-Sanigorski A, McCabe M, Mavoa H, Moodie M, Utter J, Bell C, Malakellis M, Mathews L: Reduction in overweight and obesity from a 3-year community-based intervention in Australia: the ‘It’s Your Move!‘project. Obesity Reviews 2011, 12(s2):20-28.
6	Nevada Moves Day (Bungum et al., 2014) [26]	**Bungum TJ,** Clark S, Aguilar B: The effect of an active transport to school intervention at a suburban elementary school. American Journal of Health Education 2014, 45(4):205-209.
7	Ride2School (Crawford &Garrard, 2013) [7]	**Crawford S,** Garrard J: A combined impact-process evaluation of a program promoting active transport to school: understanding the factors that shaped program effectiveness. Journal of Environmental and Public Health 2013, 2013.
8	Safe Routes to School - Eugene (McDonald et al., 2013) [27]	**McDonald NC,** Yang Y, Abbott SM, Bullock AN: Impact of the Safe Routes to School program on walking and biking: Eugene, Oregon study. Transport Policy 2013, 29:243-248.
9	Safe Routes to School - Hawaii (Heinrich et al., 2011) [28]	**Heinrich KM,** Dierenfield L, Alexander DA, Prose M, Peterson AC: Hawai ‘i’s Opportunity for Active Living Advancement (HO ‘ĀLA): Addressing Childhood Obesity through Safe Routes to School. Hawaii Medical Journal 2011, 70(7 suppl 1):21.
10	School Travel Planning - Canada (Buliung et al., 2011) [41]	**Buliung R,** Faulkner G, Beesley T, Kennedy J: School travel planning: mobilizing school and community resources to encourage active school transportation. Journal of School Health 2011, 81(11):704-712.
		1Ramanathan S, O’Brien C, Faulkner G, Stone M: Happiness in Motion: Emotions, Well-Being, and Active School Travel. Journal of School Health 2014, 84(8):516-523.
		Mammen G, Stone MR, Faulkner G, Ramanathan S, Buliung R, O’Brien C, Kennedy J: Active school travel: an evaluation of the Canadian school travel planning intervention. Preventive Medicine 2014, 60:55-59.
11	School Travel Plans - New Zealand (Hinckson et al., 2011) [40]	**Hinckson EA,** Garrett N, Duncan S: Active commuting to school in New Zealand children (2004-2008): a quantitative analysis. Preventive Medicine 2011, 52(5):332-336.
		Hinckson EA, Badland HM: School travel plans: Preliminary evidence for changing school-related travel patterns in elementary school children. American Journal of Health Promotion 2011, 25(6):368-371.
12	Stockholm County Implementation (Elinder et al., 2012) [35]	**Elinder LS,** Heinemans N, Hagberg J, Quetel A-K, Hagströmer M: A participatory and capacity-building approach to healthy eating and physical activity– SCIP-school: a 2-year controlled trial. International Journal of Behavioral Nutrition and Physical Activity 2012, 9(1):1.
13	Traveling Green (McMinn et al., 2012) [37]	**McMinn D,** Rowe DA, Murtagh S, Nelson NM: The effect of a school-based active commuting intervention on children’s commuting physical activity and daily physical activity. Preventive Medicine 2012, 54(5):316- 318.
		McMinn D, Rowe DA, Murtagh S, Nelson NM: The Strathclyde Evaluation of Children’s Active Travel (SE-CAT): study rationale and methods. BMC Public Health 2011, 11(1):1.
15	Walking School Bus - Houston (Mendoza et al., 2011) [30]	**Mendoza JA,** Watson K, Baranowski T, Nicklas TA, Uscanga DK, Hanfling MJ: The walking school bus and children’s physical activity: a pilot cluster randomized controlled trial. Pediatrics 2011, 128(3):e537-e544.
		Mendoza JA, Watson K, Chen T-A, Baranowski T, Nicklas TA, Uscanga DK, Hanfling MJ: Impact of a pilot walking school bus intervention on children’s pedestrian safety behaviors: A pilot study. Health & Place 2012, 18(1):24- 30.
		Oreskovic NM, Blossom J, Robinson AI, Chen ML, Uscanga DK, Mendoza JA: The influence of the built environment on outcomes from a “walking school bus study”: a cross- sectional analysis using geographical information systems. Geospatial Health 2014, 9(1):37.
16	Walking School Bus - Missouri (Sayers et al., 2012) [31]	**Sayers SP,** LeMaster JW, Thomas IM, Petroski GF, Ge B: A Walking School Bus program: impact on physical activity in elementary school children in Columbia, Missouri. American Journal of Preventive Medicine 2012, 43(5):S384-S389.

## Additional file

Additional file 1: Active Transport to School Database Search. (DOC 66 kb)

## Abbreviations

ALBD: Active Living by Design
AST: Active School Travel
BMI: Body mass index
ECAC: Ecological and Cognitive Active Commuting
EPHPP: Effective Public Health Practice Project
HE: Health eating
PA: Physical activity

## Footnotes

1 It is noteworthy that six interventions have multiple published papers. As the EPHPP was designed to assess studies rather than interventions [20], and in order to minimise the discrepancies between papers reporting different components of interventions, only one paper reporting information relevant to the EPHPP framework was selected for each intervention. Excluded papers did not provide any information that would affect the EPHPP rating of the study. The papers used in the EPHPP assessment are marked in the Appendix.
