# Supplementary material for: Promoting active travel to school: a systematic review (2010–2016)
Source: BMC Public Health. 2017 Aug 5;17:638. doi: 10.1186/s12889-017-4648-2 (PMC5545094; doi:10.1186/s12889-017-4648-2)
Supplement: Additional file 1: — Active Transport to School Database Search. (DOC 66 kb) [file 12889_2017_4648_MOESM1_ESM.doc]

**Active Transport to School Database Search**

**Databases to search:**

EBSCO (All Databases)✓; Emerald✓; Ovid (All Databases)✓; Proquest (All Databases)✓; Sciencedirect✓; Taylor & Francis✓; Web of Science (All Databses)✓.

Keywords:

active transport* OR active travel*

intervention* OR Randomi?ed Controlled Trial OR evaluation OR trial OR campaign* OR program* OR study OR studies

child* OR adolescent* OR parent* OR youth OR student* OR pupil*

school*

No Date Specified

**EBSCO - All Databases on [AB, TI, SU] Scholarly Journals**

Abstract (AB) = 122

Title (TI) = 5

Subject (SU) = 0

**Total Results = 122 (Duplicates removed by system)**

**Emerald Search on [AB, TI, KW]**

AB = 4

TI = 0

KW = 0

**Total Results = 4**

**Proquest All Databases Search on [AB, TI, SU] Scholarly Journals Peer Reviewed Don’t keep duplicates**

**AB = 241**

**Ti = 7**

**Su = 13**

**Total Results = 261**

**Ovid [All databases]**

**Ab = 286**

**Keywords Heading = 0**

**Su = 0**

**Ti = 0**

**MeSH Subject Heading = 0**

**Total Results = 286**

**Sciencedirect Search on [Title, Abstract, Keyword]**

TITLE-ABSTR-KEY(**active transport* OR active travel***) and TITLE-ABSTR-KEY(**intervention* OR Randomi?ed Controlled Trial OR evaluation OR trial OR campaign* OR program* OR study OR studies**) and TITLE-ABSTR-KEY(**child* OR adolescent* OR parent* OR youth OR student* OR pupil***) and TITLE-ABSTR-KEY(**school***)

**Total Results = 2**

**Taylor & Francis Search on [Article Title, Publication Title, Keywords, Abstract]**

(active transport* OR active travel*) AND (intervention* OR Randomi?ed Controlled Trial OR evaluation OR trial OR campaign* OR program* OR study OR studies) AND (child* OR adolescent* OR parent* OR youth OR student* OR pupil*) AND (school*) [“AND” must be capital]

Abstract= 129

Article Title= 1

Publication Title= 0

Keywords= 0

**Total Results = 130**

**Web of Science [Core Database] Search on [TS(Topic) TI (Title)]**

Topic (TS) = 567

Title (TI) = 9

**Total Results** **= 576**

**Grand Total = 1381 records**

**Total After Duplicates Removed = 756**

**Active Transport to School Database Search**

**Databases to search:**

EBSCO (All Databases)✓; Emerald✓; Ovid (All Databases)✓; Proquest (All Databases)✓; Sciencedirect✓; Taylor & Francis✓; Web of Science (All Databses)✓.

Keywords:

active transport* OR active travel*

intervention* OR Randomi?ed Controlled Trial OR evaluation OR trial OR campaign* OR program* OR study OR studies

child* OR adolescent* OR parent* OR youth OR student* OR pupil*

school*

Jan 2015 to Feb 2016

**EBSCO - All Databases on [AB, TI, SU] Scholarly Journals**

Abstract (AB) = 27

Title (TI) = 0

Subject (SU) = 0

**Total Results = 27 (Duplicates removed by system)**

**Emerald Search on [AB, TI, KW]**

AB = 2

TI = 0

KW = 0

**Total Results = 2**

**Proquest All Databases Search on [AB, TI, SU] Scholarly Journals Peer Reviewed Don’t keep duplicates**

**AB = 26**

**Ti = 0**

**Su = 0**

**Total Results = 26**

**Ovid [All databases]**

**Ab = 36**

**Keywords Heading = 0**

**Su = 0**

**Ti = 0**

**MeSH Subject Heading = 0**

**Total Results = 36**

**Sciencedirect Search on [Title, Abstract, Keyword]**

TITLE-ABSTR-KEY(**active transport* OR active travel***) and TITLE-ABSTR-KEY(**intervention* OR Randomi?ed Controlled Trial OR evaluation OR trial OR campaign* OR program* OR study OR studies**) and TITLE-ABSTR-KEY(**child* OR adolescent* OR parent* OR youth OR student* OR pupil***) and TITLE-ABSTR-KEY(**school***)

**Total Results = 0**

**Taylor & Francis Search on [Article Title, Publication Title, Keywords, Abstract]**

(active transport* OR active travel*) AND (intervention* OR Randomi?ed Controlled Trial OR evaluation OR trial OR campaign* OR program* OR study OR studies) AND (child* OR adolescent* OR parent* OR youth OR student* OR pupil*) AND (school*) [“AND” must be capital]

Abstract= 0

Article Title= 0

Publication Title= 0

Keywords= 0

**Total Results = 0**

**Web of Science [Core Database] Search on [TS(Topic) TI (Title)]**

Topic (TS) = 81

Title (TI) = 0

**Total Results** **= 81**

**Grand Total = 172 records**

**Total After Duplicates Removed = 101**
